# Interpersonal Movement Synchrony Responds to High- and Low-Level Conversational Constraints

**DOI:** 10.3389/fpsyg.2017.01135

**Published:** 2017-07-28

**Authors:** Alexandra Paxton, Rick Dale

**Affiliations:** ^1^Institute of Cognitive and Brain Sciences, University of California, Berkeley Berkeley, CA, United States; ^2^Berkeley Institute for Data Science, University of California, Berkeley Berkeley, CA, United States; ^3^Cognitive and Information Sciences, University of California, Merced Merced, CA, United States

**Keywords:** interpersonal coordination, synchrony, joint action, conversation, movement dynamics, cross-recurrence quantification analysis, working memory, dual-task performance

## Abstract

Much work on communication and joint action conceptualizes interaction as a dynamical system. Under this view, dynamic properties of interaction should be shaped by the context in which the interaction is taking place. Here we explore *interpersonal movement coordination* or *synchrony*—the degree to which individuals move in similar ways over time—as one such context-sensitive property. Studies of coordination have typically investigated how these dynamics are influenced by either *high-level constraints* (i.e., slow-changing factors) or *low-level constraints* (i.e., fast-changing factors like movement). Focusing on nonverbal communication behaviors during naturalistic conversation, we analyzed how interacting participants' head movement dynamics were shaped simultaneously by high-level constraints (i.e., conversation type; friendly conversations vs. arguments) and low-level constraints (i.e., perceptual stimuli; non-informative visual stimuli vs. informative visual stimuli). We found that high- and low-level constraints interacted non-additively to affect interpersonal movement dynamics, highlighting the context sensitivity of interaction and supporting the view of joint action as a complex adaptive system.

## 1. Introduction

Human interaction is a complex and dynamic process. From the subtle modulation of speech to the dynamic displacement of the body in posture or gesture, humans must fluidly organize behavior in time across multiple modalities to interact effectively with one another. Contributing to the ongoing debate about the underlying mechanisms of interpersonal processes (for reviews, see Brennan et al., 2010; Dale et al., 2013; Barr, 2014; Paxton et al., 2016), we here build on previous work (Paxton et al., 2016) to propose that *context* is critical for understanding how interaction unfolds. By using advances in wearable technology (Paxton et al., 2015) to manipulate task parameters during an interactive experiment, we explore the influence of context on dynamics of body movement during conversation and turn to a particular theoretical framework to help understand it: *dynamical systems theory* (*DST*).

From biomes to hurricanes, many physical and biological systems are recognized as *complex dynamical systems*. These systems exhibit what are called *emergent properties*—that is, characteristic behaviors that emerge not by instructions from some top-down controller but as a function of local interactions among the component parts within given contextual pressures. A famous example of this is the so-called “butterfly effect.” This principle suggests that subtle factors in a present context may cascade into larger effects, which themselves serve as a context that constrains ongoing behavior (e.g., Lorenz, 1963). While it began in the physical and mathematical sciences, DST has become a powerful lens for understanding human behavior and cognition as well (Barton, 1994; Mathews et al., 1999).

DST—along with other complexity sciences (cf. Mathews et al., 1999)—provides a conceptual and analytic framework to capture the context-sensitive, soft-assembled, emergent properties of cognitive, behavioral, and affective phenomena. Though its influence is still growing in psychology more broadly, DST principles and analyses have led to novel insights into such phenomena as reading (e.g., Van Orden and Goldinger, 1994), gaze (e.g., Engbert et al., 2005), cultural evolution (e.g., Kenrick et al., 2003), general cognitive function (e.g., Van Orden et al., 2003), and more. DST—and, more specifically, a branch called synergetics (Haken, 1977)—has significantly influenced the understanding of self-organizing principles in cognition (e.g., Haken, 1990; Stadler and Kruse, 1990; Haken and Portugali, 1996).

Increasingly, cognitive scientists interested in social phenomena are recognizing the value of DST to understanding human interaction (e.g., Vallacher et al., 2002; Coleman et al., 2007). Within this area, DST may be uniquely equipped to explore *interpersonal coordination*—the idea that individuals influence one another's behavior, cognition, and emotion as a result of their interaction. By shifting analysis away from the individual and conceptualizing the *dyad* as the focus of analysis, we can begin to explore the behavioral, cognitive, and emotional dynamics that emerge from the contextual pressures constraining the dyadic system—like the specific task or type of conversation in which the dyad is engaging.

Interpersonal coordination has been an increasingly influential way to capture interpersonal dynamics over the last few decades (Condon and Sander, 1974). This phenomenon has been studied under a variety of names—like accommodation, alignment, the “chameleon effect,” contagion, coordination, coupling, mimicry, synchrony, and synergy^1^. Interestingly, the idea that coordination and other behaviors are adaptive in the DST sense extends to even some of the earliest works in this domain (Sander, 1975).

Within interpersonal coordination research, the *interpersonal synergies* perspective has perhaps the strongest connection to DST ideas (Riley et al., 2011). Historically, most work on interpersonal coordination has tended to be characterized by what we have called a “more is better” perspective (see Abney et al., 2015). This perspective holds that individuals tend to become more similar over time as a result of their interaction and that this increased similarity tends to be better for a variety of interaction outcomes (e.g., Pickering and Garrod, 2004).

However, the interpersonal synergies perspective posits that interpersonal dynamics are fundamentally shaped by a variety of factors that exert pressure on the interpersonal system. Under this view, interacting participants will not necessarily become uniformly more similar over time. Instead, different contextual factors—like interactants' relationship, goals, physical or perceptual environment, affordances (in the Gibsonian sense; e.g., Gibson et al., 1999) and conversation type—will lead to different configurations of behavioral channels (e.g., Fusaroli and Tylén, 2016).

Inspired by research on DST, we have elsewhere proposed a classification system for different components of an interaction (Paxton et al., 2016), dividing the influences on communication dynamics into *top-level and bottom-level systems*. *Top-level systems* function at a lower frequency, change over longer timescales, and tend to have fewer degrees of freedom; *bottom-level systems*, by contrast, function at a higher frequency, can change over very short timescales, and tend to have more degrees of freedom^2^. Examples of top-level systems would include conversational contexts and interpersonal relationships; bottom-level systems would include body movement or phonetics.

Studies of coordination often focus on only one of these systems at a time—like how coordination influences rapport (e.g., Hove and Risen, 2009) or how perceptual information influences coordination (e.g., Richardson et al., 2007a). In this paper, we explore how simultaneous constraints on both systems influence coordination: *high-level contextual constraints* (i.e., those affecting the overarching top-level systems) and *low-level contextual constraints* (i.e., those affecting the rapidly changing bottom-level systems).

Approaching nonverbal social behavior during conversation from the synergies perspective, the present study focuses on how high- and low-level contextual constraints can change interpersonal coordination over time in naturalistic interaction. Specifically, we explored how conversation type—whether argument or a friendly conversation—and perceptual information—either informative or noisy perceptual signals^3^—altered coordination of interacting participants' head movements. We proposed four hypotheses, guided by previous findings.

Keeping with our earlier work (Paxton and Dale, 2013a,b), we use “coordination” as a general term for the idea that individuals affect one another's behavior over time as a result of their interaction. We use “synchrony” as a specific case of coordination: Interacting individuals are *synchronized* to the extent that they tend to exhibit the same behavior at the same time. Although we do not explore time-locked phase synchrony here (cf. Richardson et al., 2007a), we use time series analyses to quantify whether interacting individuals generally tend to behave similarly in time.

*H*_1_*: Overall, head movement will be synchronized*.

Previous work suggests that interacting individuals' gross body movements (Nagaoka and Komori, 2008; Paxton and Dale, 2013a) and head movements specifically (Ramseyer and Tschacher, 2014; Paxton et al., 2015) become more similar over time as a result of their interaction. Therefore, we expect that we will find that participants' head movement will be synchronized. That is, we anticipate that participants will be more likely to move (or not move) their heads at the same time than not.

*H*_2_*: Dynamics of nonverbal communication signals will be sensitive to conversation type (as a high-level contextual constraint)*.
*H*_2*A*_*: Argument—compared to friendly conversation—will decrease head movement synchrony*.

Mounting evidence suggests that coordination dynamics are sensitive to high-level contextual perturbations (Miles et al., 2011), including conversation type (Paxton and Dale, 2013a; Abney et al., 2014; Main et al., 2016). Despite some exceptions—for example, when analyzing gaze patterns (Paxton et al., under review) and when discussing assigned (rather than personally held) beliefs (Tschacher et al., 2014)—conflict has been found to decrease interpersonal synchrony (Paxton and Dale, 2013a; Abney et al., 2014). We therefore expect to find some difference in movement synchrony between the two conversation types (*H*_2_); directionally, we expect that argument will decrease synchrony (*H*_2*A*_).

*H*_3_*: Dynamics of nonverbal communication signals will be sensitive to perceptual information (as a low-level contextual constraint)*.
*H*_3*A*_*: Changing visual information interpreted as noise—rather than a meaningful signal to be remembered—will increase head movement synchrony*.

Low-level contextual constraints—like perceptual information—have been relatively less studied in coordination research. This may have been due to limitations in previous experiment tools: Any perturbations to the dyadic system have had to expose both participants in a naturalistic, face-to-face interaction to the same environmental stimulus. A previous study found that holding a conversation over loud ambient noise—as compared with an otherwise silent room—led to an increase in head movement synchrony (Boker et al., 2002). This supports the idea that interpersonal coordination may serve to boost the “signal” in communication within the “noise” of the environment (Richardson and Dale, 2005; Shockley et al., 2009).

Although the concept of “information” has a variety of meanings within cognitive science, we here simply mean that the signal is imbued by the participant as having relevance to some task. For the present study, this is not a signal that is relevant to the conversation itself but to another memory task. It is contrasted with signals in the environment that are not directly relevant to any task at hand—signals that we may call “noise.” Crucially, in the current study, both sets of stimuli are otherwise identical, allowing us to disentangle the effects of the stimulus itself and the information imbued in the signal by the interlocutor.

The current study extends previous work to see whether visual “noise” can serve the same function as auditory noise—boosting synchrony and, possibly, comprehension. We hypothesize that nonverbal communication signals will respond to low-level contextual constraints or perturbations (*H*_3_). Directionally, we expect that noise will increase synchrony (*H*_3*A*_).

*H*_4_*: Dynamics of nonverbal communication signals will be non-additively sensitive to conversation type (as a high-level contextual constraint) and to perceptual information (as a low-level contextual constraint)*.

While previous studies have focused on the effects of either high-level constraints (e.g., Miles et al., 2011; Paxton and Dale, 2013a; Abney et al., 2014; Main et al., 2016) or low-level constraints (e.g., Boker et al., 2002; Richardson et al., 2007a), we are unaware of any studies to date that have combined the two. We see our work as providing a vital step in the exploration of interaction and coordination under the DST perspective: If communication is a dynamical system, we would expect to see that behavior is context sensitive and does not uniformly react to all constraints (cf. Riley et al., 2011; Paxton et al., 2016). Therefore, we hypothesize that head movement synchrony will be non-additively sensitive to both high- and low-level constraints; however, as the first such study of these simultaneous dynamics (of which we are aware), we do not have a directional hypothesis.

## 2. Methods

### 2.1. Participants

Forty-two undergraduate students from the University of California, Merced participated as 21 dyads. Dyads were created as participants individually signed up for experiment appointments per their own availability through the online subject pool system. Each participant received course credit in return for participation. By chance, dyads included some pairs of women (*n* = 9; 43%), some pairs of men (*n* = 3; 14%), and mixed-gender pairs (*n* = 9; 43%) according to participants' self-reported gender identities. Participants in 2 dyads reported being acquainted with one another prior to the experiment (10%).

Additional dyads—not included in the counts above—participated but were not analyzed here. Two (2) additional dyads were excluded due to lack of conflict in the argumentative conversation, as we have done in previous work using a similar paradigm (Paxton and Dale, 2013a).

We also experienced technical difficulties with the servers running our data collection program for a number of additional dyads. In order to be included in the present analysis, each participant in the dyad must have had recorded movement data for at least 4.5 min (including the calibration period; see Section 2.3) of each of the two conversations described in Section 2.2. In an additional 21 dyads (not included in the counts above), the server failed to record the minimum 4.5 min of movement data for at least 1 of the 2 participants in at least 1 of the 2 conversations. This occurred because the program used to run the data collection software prioritizes fidelity of the connection to the data collection server above all (see Paxton et al., 2015); any perturbation of that connection causes the program to be terminated. However, until the point of termination, data were continuously and regularly sampled.

For example, assume that participants A and B are participating in the experiment. For the first 3 min, the movements of participants A and B are sampled regularly (according to Section 2.2). At minute 4 of the first conversation, participant A's connection to the server is perturbed, causing the server to disconnect participant A's movement tracker. Participant A's regularly sampled data for the first 3 min are saved, but no further data for participant A are recorded, although the conversation continues as usual. Participant B's tracker, however, remains connected to the server, and after being regularly sampled for the rest of the 8-min conversation, participant B's data are saved. Even if all 8 min of movement data were successfully saved for both participants in the second conversation, this dyad would be excluded from our analysis. Although participant A has an unbroken 3-min movement time series from the first conversation and an unbroken 8-min movement time series from the second conversation, this dyad would not have the minimum 4.5 min of movement data for *both* participants in *both* conversations.

Although this prioritization led us to discard a number of dyads due to insufficient data, it also allowed us to ensure that the behavior of the included dyads were continuously and regularly sampled during the experiment—leading to very few missing samples in the included dyads. We chose this cutoff prior to analysis and did not explore other thresholds for inclusion.

### 2.2. Materials and procedure

This study was carried out in accordance with the recommendations of Institutional Review Board of the University of California, Merced, with written informed consent from all subjects. All subjects gave written informed consent in accordance with the Declaration of Helsinki. The protocol was approved by the Institutional Review Board of the University of California, Merced.

As noted below (see Section 2.2.2), the informed consent process did not give any foreknowledge of the specific phenomenon (i.e., similarity of movement), conversation prompts or topics, nor the hypotheses of the study. Data were collected by research assistants blind to study hypotheses for 20 of the 21 dyads; data for the remaining (1) dyad was collected by the first author. The first author also assisted in data collection for 4 dyads.

#### 2.2.1. Experiment design

The experiment had one within-dyads and one between-dyads element. Conversation type was a within-dyads condition and was adapted from Paxton and Dale (2013a): Each dyad had one argumentative conversation and one affiliative conversation. Conversation order was counterbalanced (randomly assigned) to prevent order effects. For the between-dyads condition, each dyad was randomly assigned to a “noise” (*n* = 9; 43%) or a “dual-task” (*n* = 12; 57%) condition^4^. Both conditions are described in greater detail below.

#### 2.2.2. Data collection

Upon arriving, participants were separated and led to private (semi-enclosed) areas with desks within the lab. Each was then given a series of questionnaires, including a sociopolitical opinion questionnaire. The opinion questionnaire neutrally inquired about the participant's opinion on a variety of issues (e.g., abortion, death penalty, marriage equality^5^, whether Spanish should be an official U.S. language, whether student loans should be partially forgiven by the U.S. government). The participant responded to each question in a brief, open-ended response area and by indicating opinion strength on a 1 *(feel very weakly)* to 4 *(feel very strongly)* scale.

After both participants completed the questionnaires, they were brought together in a small, private space. Participants were seated facing one another in stationary chairs approximately 0.97 m (3.17 feet) apart (measured at the front legs). Participants were told that they would be having “two conversations for about 8 min each” for a study “about how people hold conversations,” but no information about the nature or emotional valence of the prompts was given. (If asked, participants were told that they would be given the conversation topic immediately before beginning each conversation.) The experimenter then told the participants to take a few minutes to introduce themselves to one another while the experimenter stepped outside of the room to complete some last-minute paperwork before beginning the experiment.

The experimenter then left the room for approximately 3 min. Unknown to the participants, the experimenter spent this time comparing the two participants' opinion surveys to identify up to 3 topics for which participants (a) wrote the most differing opinions and (b) indicated the strongest opinions. We refer to these as “candidate argumentative topics” below.

After 3 min, the experimenter re-entered the room and gave each participant a Google Glass (Alphabet, Inc.), a piece of wearable technology worn like glasses that features a small quartz screen over the wearer's right eye and an on-board processor on the wearer's right temple. The experimenter then explained the device to the participants, adjusted the Glass (as necessary) to fit each participant, and tested to ensure that each participant could fully see the screen. (For complete fitting procedure, see Paxton et al., 2015.) Participants were reminded that they would be having “a couple of conversations about different topics” and that they would “[be given] the topic for each conversation right before [they] start.” They were told that the Google Glass would be “recording information about the conversation,” but the nature of the recorded information was not described in detail to avoid drawing participants' attention to their head movements^6^.

Each Google Glass ran the PsyGlass program (Paxton et al., 2015). Once initialized, each participant's PsyGlass program randomly generated a screen color at 1Hz (i.e., 1 color per s), with a 0.9 probability of generating a blue screen and a 0.1 probability of generating a red screen. (If *color*_*t*_ were the same as *color*_*t*+1_, the screen did not flicker or otherwise indicate that it was refreshing). PsyGlass also recorded participants' head movements by measuring the three-dimensional accelerometer data (i.e., *x, y, z* axes, with the origin point set as the position at the time of initialization) at 250 Hz (i.e., 1 sample per 4 ms), which were transmitted to and stored on the experiment server at 4 Hz (i.e., 1 transmission per 250 ms). All code for the program can be downloaded from the GitHub (GitHub, Inc.; http://www.github.com) repository for PsyGlass (http://www.github.com/a-paxton/PsyGlass).

#### 2.2.3. Task condition (between-dyads)

Before initializing PsyGlass on each participant's device, both participants were given instructions about their between-dyads condition. All dyads—across both task conditions—were exposed to the same stimuli through PsyGlass, the same red-and-blue screens. The two conditions differed only in the interpretation or significance of the colored screens. In the *noise* condition, the dyad was told that the flashing screens were a result of a bug in the program and that participants should have conversations as normal. In the *dual-task* condition, the dyad was told to remember the number of times that the screen turned red while having their conversation and that they would be asked to write that number down after the conversation was finished (similar to the oddball paradigm; Squires et al., 1975). After answering any participant questions, the PsyGlass program was initialized.

#### 2.2.4. Conversation type (within-dyads)

Again, each dyad held 2, 8-min conversations—one affiliative conversation and one argumentative conversation. In both cases, participants were instructed to stay on the assigned topics or on topics very similar to the assigned topic. After assigning each prompt, the experimenter remained seated behind a computer outside of the participants' immediate peripheral vision, surreptitiously monitoring the conversation.

The affiliative prompt was identical across all dyads, asking them to discuss media that they both enjoyed, find something that they both enjoyed, and talk about why they liked it. The goal of the affiliative prompt was to emphasize similarity and engender rapport between participants.

The argumentative prompt relied on the candidate argumentative topics identified from the opinion surveys. The prompt asked participants to discuss their views on the top-rated candidate topic (again using neutral phrasing) and asked participants to “try to convince one another of [their] opinions.” If the conversation stopped altogether or shifted away from being argumentative in nature (e.g., if both participants came to a consensus), the next highest-rated candidate topic was assigned. If the second candidate topic again failed to produce sustained argumentative conversation, the third candidate topic was assigned.

After initializing PsyGlass for the first time, the prompt for the first (randomly assigned) conversation type was given. After 8 min of conversation, the experimenter informed the participants that their conversation was over, and PsyGlass was terminated. Participants then removed their Google Glass and were led to their private desks to complete two brief questionnaires about the conversation (not analyzed here), including—for dual-task condition dyads—the number of times they had seen a red screen. Once both participants had completed the questionnaires, participants were brought back to the joint space and re-fitted with the Google Glass. After ensuring that both participants could again see the entire screen, PsyGlass was initialized, and the remaining prompt was given.

Participants were not given any foreknowledge about the topics or type of conversation before being assigned the relevant prompt. That is, if participants were assigned to have the affiliative conversation first, they had no knowledge that their second conversation would have an argumentative prompt; the same applied if the participants had had the argumentative conversation first.

### 2.3. Data preparation

Each participant produced one movement time series for each conversation. The time series captured timestamped accelerometer values along *x, y, z* axes. After applying an anti-aliasing zero-phase fourth-order Butterworth filter, we downsampled the data to 10 Hz, a sampling rate similar to those utilized in our previous movement coordination work (Paxton and Dale, 2013a; Abney et al., 2015; Paxton et al., 2015). We transformed these three-dimensional values into a single value for acceleration at each time point by taking the three-dimensional Euclidean distance of the time series. We then applied a smoothing zero-phase second-order Butterworth filter to the acceleration signal for each participant.

We then trimmed the movement data to remove the time between the PsyGlass initialization and the beginning of the conversation data. Immediately before beginning each conversation (i.e., after having been given the appropriate prompt), participants were asked to produce a brief bout of high-velocity head movement (nodding and shaking their heads rapidly). This was done under the guise of “initializing the program” but was used as a marker for the beginning of the conversation data.

Because each dyad took 60–120 s to test PsyGlass and hear the conversation prompt, we used derivatives of acceleration to identify the latest moment of intense movement by both participants during that window. We explored both acceleration's first-order (jerk) and second-order (jounce) derivatives to identify possible markers. The cutoff points identified by jerk and jounce were significantly correlated, *r* = 0.62, *t*_(40)_ = 4.94, *p* < 0.0001. However, because jounce produced more conservative (i.e., later) estimates of cutoff times, we used jounce.

Cutoff times for each conversation were created at the dyad level. For each participant in each dyad, we identified the time of largest jounce in the first 60–120 s of the conversation. We then chose the more conservative (i.e., later) cutoff point of the two participants, which we applied as both participants' cutoff points.

Finally, we truncated both participants' time series in each conversation to the shorter of the two lengths, if they were not already identical (e.g., due to server failure). Because PsyGlass initializes data collection simultaneously for both participants (Paxton et al., 2015), we did not need to time-align the beginning of the conversation.

After applying these cutoffs, conversations had an average of 6.54 min (range = 2.62–9.26 min) of recorded movement data. It is important to emphasize that dyads completed the full experimental conditions even when they did not have complete movement records: Connectivity issues with the experimental server only resulted in a failure to record the movement time series *after* the perturbation to the data collection server occurred. We find it important to note because if participants had experienced different experimental conditions (e.g., if some had held only a 5-min first conversation while others had held an 8-min first conversation), we could not infer that the intended manipulations (i.e., conversation type and task condition) were the cause of any effects, rather than any of the unintended conditions (e.g., shorter conversations).

### 2.4. Data analysis

We measured coordination by combining *cross-recurrence quantification analysis* (*CRQA*) and *growth curve analysis* (*GCA*). This combination allows us to quantify the amount of moment-to-moment coordination occurring between interacting participants, along with longer-scale trends. We describe these techniques briefly below, but a more detailed explanation of the benefits of using CRQA and GCA together can be found in Main et al. (2016)^7^. We then used a linear mixed-effects model to analyze the resulting data.

#### 2.4.1. Cross-recurrence quantification analysis

CRQA is an outgrowth of recurrence quantification analysis (RQA), a nonlinear time series analysis that captures the structure and patterns of states visited by a single dynamical system over time (Eckmann et al., 1987). CRQA extends RQA by capturing the amount to which two different systems co-visit similar states in time and has become a staple for analyzing human data from a dynamical systems perspective (e.g., Shockley et al., 2003; Dale and Spivey, 2006; Richardson et al., 2007b; Gorman et al., 2012; Anderson et al., 2013; Fusaroli et al., 2014; Vallacher et al., 2015). Detailed explanations of CRQA and its applications in a variety of settings are available in Marwan et al. (2007), Coco and Dale (2014), and Main et al. (2016).

In our case, CRQA allows us to quantify when two participants moved in similar ways during conversation. Unlike studies of more rhythmic movements (e.g., tapping to a metronome), head movement dynamics during conversation comprise both periodic (e.g., underlying postural sway) and non-periodic (e.g., nodding intermittently during conversation) components—leaving phase-coupling analyses (e.g., Richardson et al., 2007a) less suitable for our current purposes. We chose CRQA as a method that does not assume or require periodicities and that can be more resilient to the noise inherent in a new method (i.e., measuring interpersonal dynamics with head-mounted accelerometers in Google Glass).

Current best practices for continuous CRQA include reconstructing the phase space for each pair of signals using time-delay embedding (Shockley et al., 2003; Riley and Van Orden, 2005) and then calculating recurrent points by identifying the radius size at which overall recurrence rate (RR) of the plot is equal to 5% (cf. Marwan et al., 2007; Konvalinka et al., 2011). More detailed information on phase space reconstruction and embedding are available from March et al. (2005) and Iwanski and Bradley (1998). We follow these best practices to calculate CRQA for each conversation of each dyad^8^. The parameters for each dyad are available in the OSF and GitHub repositories for the project (see Section 3).

CRQA was implemented in R (R Core Team, 2016) using the crqa library (Coco and Dale, 2014). We obtained the *diagonal recurrence profile* (DRP) for each conversation of each dyad. The DRP captures how much coordination occurs within a “window” of relative time between participants. Here, we target a window of ±5 s, consistent with previous work on body movement coordination generally (Paxton and Dale, 2013a) and head movement specifically (Ramseyer and Tschacher, 2014). With a sampling rate of 10 Hz, this creates a window of interest of ±50 samples. Intuitively, the DRP can be read much like a cross-correlation profile (Paxton and Dale, 2013a), with some differences. (For more on the differences between DRPs and cross-correlation profiles, see Main et al., 2016.)

Essentially, the DRP allows us to explore similarities in patterns of movement that are independent of *absolute* time while revealing patterns of *relative* time. The DRP captures leading and following patterns along with simultaneous movement. In other words, we are able to use DRPs to see, at any given time in the conversation, whether participants are more likely to be moving in similar ways (i.e., higher *rate of recurrence* or RR) or in dissimilar ways (i.e., lower *rate of recurrence* or RR).

Because both participants will have the same length time series (because of identical sampling rates within the experiment), Participant A and Participant B will both have samples for all time points, *t*. The DRP compares Participant A's head movement at *t* with Participant B's head movement at *t* − 50, …, *t*, …, *t* + 50. When Participant B's *t* < 0, the DRP captures the degree to which Participant B leads the movement state for Participant A at *t*; when Participant B's *t* > 0, the DRP captures the degree to which Participant A at *t* leads the movement state for Participant B. When we compare Participant A's movement at *t* with Participant B's movement at *t*, the DRP captures the amount to which both participants engaged in movement at the same time. The DRP also captures the reverse—comparing Participant B's head movement at *t* with Participant A's head movement at *t* − 50, …, *t*, …, *t* + 50.

#### 2.4.2. Growth curve analyses

GCA is a time series analysis used to quantify the degree to which changes over time can be best described by various orthogonal polynomials (Mirman et al., 2008). Rather than assuming that data are described by a linear relationship, GCA determines how well the data are fit by polynomial relationships (e.g., linear, quadratic, cubic) and disentangles the contribution of each polynomial independently. In the current analysis, we focus only on the first- and second-order orthogonal polynomials.

In other words, GCA allows us to distinguish how much the linear and quadratic forms *separately* contribute to the overall shape of the data. As a result, GCA is a powerful technique for quantitatively comparing DRPs, allowing us to explore leading/following patterns (with the linear lag term) and coordination patterns (with the quadratic lag term).

#### 2.4.3. Model specifications

All data analysis was performed in R (R Core Team, 2016). Using the lme4 library (Bates et al., 2015), we created a linear mixed-effects model to quantify the effects of linear lag (LL; leading/following) and quadratic lag (QL; coordination) with conversation type (within-dyads; dummy-coded: affiliative [0] or argumentative [1]) and task (between-dyads; dummy-coded: dual-task [0] or noise [1]) on head movement recurrence rate (RR). Dyad and conversation number were included as random intercepts; for both random intercepts, we included the maximal random slope structure that permitted model convergence using backwards selection per current best practices for linear mixed-effects models (Barr et al., 2013). Compared against the random-intercepts-only model, the maximal model justified by the data better fits the data; these results are provided in the supplemental repositories for the project (see Section 3).

As discussed below (see Section 3), our data and analysis materials—including code with the precise specifications for all models—are freely available in public repositories for the project. For interested readers, we here provide the single-equation mathematical form of our linear mixed-effects model using Barr et al.'s (2013) conventions:

$$\begin{array}{rcl} RR_{dt} & = & \beta_{0}+D_{0d}+N_{0d}+\left(\beta_{1}+D_{1d}+N_{1d}\right)c_{d}+\beta_{2}k_{d}\\ +\left(\beta_{3}+D_{3d}+N_{3d}\right)l_{dt}+\left(\beta_{4}+D_{4d}+N_{4d}\right)q_{dt}\\ +\beta_{5}c_{d}k_{d}+\beta_{6}l_{dt}q_{dt}+\beta_{7}c_{d}l_{dt}+\beta_{8}k_{d}l_{dt}\\ +\left(\beta_{9}+D_{9d}+N_{9d}\right)k_{d}c_{d}l_{dt}+\beta_{10}c_{d}q_{dt}+\beta_{11}k_{d}q_{dt}\\ +\beta_{12}k_{d}c_{d}q_{dt}+\beta_{13}c_{d}l_{dt}q_{dt}+\beta_{14}k_{d}l_{dt}q_{dt}+\beta_{15}k_{d}c_{d}l_{dt}q_{dt}\\ +e_{dt} \end{array}$$

Equation (1) estimates the recurrence rate *RR* for any dyad *d* at lag *t*. It does so by estimating the global coefficients—notated as β_1,…,15_—for each fixed effect: conversation type *c*, task condition *k*, linear (i.e., orthogonal first-order polynomial) lag *l*, quadratic (i.e., orthogonal second-order polynomial) lag *q*, and all interaction terms. Random intercepts for dyad identity *D*_0_ and conversation number *N*_0_ are included. We also include the maximal slope structure that permit model convergence using backwards selection from the fully maximal model in accordance with current best practices (Barr et al., 2013). The fixed effects included in the maximal slope structure for random intercepts are noted above (β_*n*_ + *D*_*nd*_ + *N*_*nd*_).

Although we report effects of LL in the model (noted *l* above), we are cautious in interpreting them. Participants were paired by a fairly random process (i.e., by individual sign-ups for open experimental timeslots that did not allow participants to see their partner's identity) and were randomly assigned to their seat in the interaction space (i.e., by arrival time; each chair was closer to one or the other of the private questionnaire spaces). Unlike previous studies (Main et al., 2016), we had no *a priori* expectations about or reasons to expect leading/following behaviors; therefore, we refrain from deeply interpreting any LL results.

#### 2.4.4. Comparing to baseline

In keeping with recommended baselines for nonlinear analyses, we also create a baseline using a Fourier phase-randomization analysis (Theiler et al., 1992; Kantz and Schreiber, 2004). Phase randomization creates a surrogate dataset that contains the same power spectrum as the real data but differs in phases, retaining the autocorrelations of the original time series. Here, we use the nonlinearTseries package (Garcia, 2015) in R (R Core Team, 2016) to create 10 phase-randomized surrogate time series for each conversation of each dyad to provide a more robust baseline analysis. We then perform CRQA over these new time series using the same parameters as the real data. Essentially, the resulting recurrence dynamics capture the amount of similarity that emerges by chance between the two time series (in this case, interacting individuals)^9^.

In our Supplementary Materials on GitHub and the OSF (see Section 3), we also perform a baseline analysis using a *sample-wise shuffled baseline*, a more common baseline technique in interpersonal coordination research that breaks temporal correspondence between two time series by separately randomizing (or shuffling) the order of each sample from the real behavior time series (Dale et al., 2011; Louwerse et al., 2012). Although this destroys more inter-sample dependencies, the sample-wise shuffled baseline also destroys the autocorrelation of the time series. This creates a somewhat less conservative baseline, as shuffled baselines cannot strongly account for the hysteresis of the system. Because the samples are shuffled independently, the temporal dynamics of shuffled baselines through their reconstructed phase-spaces are not influenced by their previous time-steps. By retaining the autocorrelation of the individual time series in the phase-randomization surrogate analysis, we are able to account for the chance that two individual time series might “live” in similar regions for some amount of time simply due to their own dynamics, rather than the influence of the other time series.

We provide the results from analyses using the sample-wise shuffled baseline in our Supplementary Materials (see Section 3). The results are highly similar to those performed against the phase-randomization baseline, although our results suggest that the phase-randomization baseline provides a more conservative metric for the amount of synchrony that might occur by chance.

## 3. Data and code sharing

We have made data and code (including code for data preparation and analysis) for the project freely available according to current best practices for data stewardship. Due to the nature of self-disclosure in the conversation data (especially in the argumentative context), we were permitted to release only limited information about each dyad: de-identified movement time series for each participant in each conversation, the dyad's assigned experimental condition, and the dyad's gender makeup.

Current best practices for open science include the sharing of data and code in public repositories (see Nosek et al., 2015; Blohowiak et al., 2016; Gewin, 2016; Kidwell et al., 2016). Two prominent venues for storing and sharing materials are the Open Science Framework (OSF; http://osf.io) and GitHub (GitHub, Inc.; https://www.github.com/). Both OSF and GitHub serve as platforms to share materials, promote community contribution, and facilitate open re-use (and re-analysis) of materials by others through appropriate attribution. Furthermore, the OSF allows researchers to “freeze” specific versions of the project—for example, at the point of publication (as we have done here)—providing a crystallized, unmodifiable snapshot of all files at that time.

All data and code for the project are freely available through our OSF repository (Paxton and Dale, 2017): https://osf.io/4yqz8/

All code can also be freely accessed through our project's GitHub repository: https://www.github.com/a-paxton/dual-conversation-constraints

## 4. Results

All analyses were performed in accordance with the model specifications described in Section 2.4.3. We here present only the standardized model, as it allows us to interpret estimates as effect sizes (see Keith, 2005). (The unstandardized model is available in the project's OSF and GitHub repositories; see Section 3.) Full standardized model results are presented in Table 1. For clarity within the text, we reference main and interaction terms in parentheses within the text so that readers can easily find the relevant values in Table 1.

**Table 1:** Results from the standardized linear mixed-effects model (implemented with lme4; Bates et al., 2015) predicting recurrence of head movement between participants (RR) with conversation (within-dyads; dummy-coded: affiliative [0] or argumentative [1]), task (between-dyads; dummy-coded: dual-task [0] or noise [1]), linear lag (LL; leading/following), and quadratic lag (QL).

**Predictor**	**Estimate**	**Std. Error**	***t*-value**	***p*-value**	**Sig**.
Conversation	−0.601	0.114	−5.288	<0.001	^***^
Task	−0.102	0.109	−0.938	0.350	
LL	0.023	0.055	0.412	0.680	
QL	0.054	0.045	1.200	0.230	
Conversation × task	0.133	0.103	1.289	0.197	
LL × QL	−0.039	0.005	−7.089	<0.001	^***^
Conversation × LL	−0.039	0.035	−1.130	0.260	
Task × LL	−0.011	0.043	−0.265	0.790	
Task × conversation × LL	−0.039	0.044	−0.878	0.380	
Conversation × QL	0.036	0.004	9.072	<0.001	^***^
Task × QL	0.019	0.040	0.480	0.630	
Task × conversation × QL	0.067	0.004	17.408	<0.001	^***^
Conversation × LL × QL	0.003	0.005	0.542	0.590	
Task × LL × QL	−0.004	0.005	−0.640	0.520	
Task × conversation × LL × QL	0.000	0.005	0.057	0.960	

The model's fixed effects alone accounted for 37% of the variance (marginal R^2^ = 0.37), while the fixed and random effects accounted for 94% of the variance (conditional R^2^ = 0.94). ^.^p < 0.10; ^*^p < 0.05; ^**^p < 0.01;

*** *p < 0.001*.

Results indeed suggested that high- and low-level constraints influence coordination dynamics—even in some unexpected ways. Contrary to our hypothesis *H*_1_, we did not find evidence of overall time-locked synchrony. Participants' head movements were, in fact, better described by a turn-taking pattern with slight leading-following dynamics (*LL* × *QL*).

**Figure 1:**
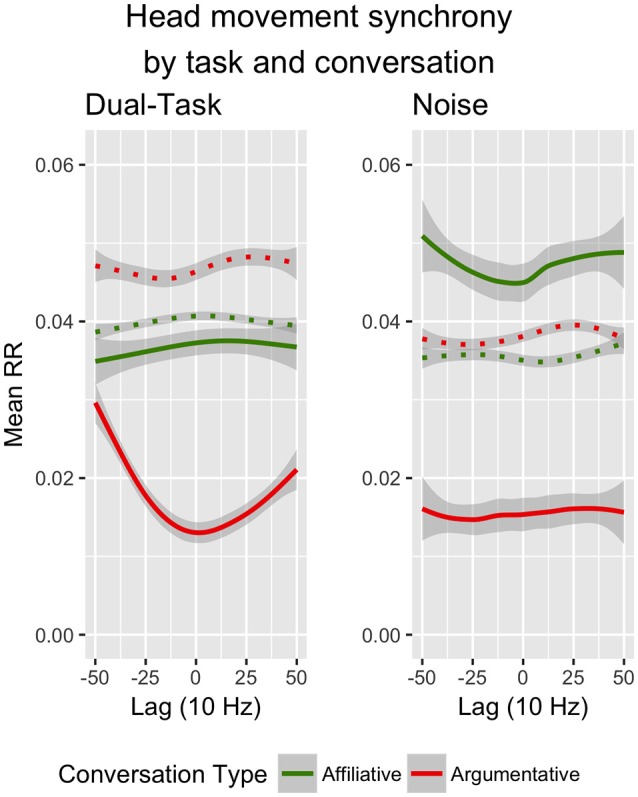
Interaction of conversation type (green = affiliative, red = argumentative), task condition (left = dual-task, right = noise), and lag (LL = slope; QL = curvature) on head movement synchrony (RR). Phase-randomized surrogate baselines are graphed in dotted lines of corresponding color. Lag is graphed in the 10 Hz sampling rate (10 samples/s). Shaded bands represent standard error. Created in R (R Core Team, 2016) with ggplot2 (Wickham, 2009).

Consistent with our hypotheses *H*_2_ and *H*_2*A*_—and replicating our previous findings (Paxton and Dale, 2013a)—we found that argument significantly decreased RR compared to affiliative conversations (*conversation*; see Figure 1). Conversation also affected moment-to-moment coupling dynamics: Recurrence during the affiliative conversations was higher but more diffuse, while recurrence in the argumentative conversation was lower and showed a distinct turn-taking pattern (*conversation* × *QL*).

Interestingly, although we hypothesized that the noise condition would increase RR compared to the dual-task condition, we did not find a significant main effect of task condition (*task*). Instead, we found that task affected the *dynamics* of coordination only in conjunction with other pressures (*task* × *conversation* × *QL*). We explored these patterns in greater depth by analyzing each of the conversation types (i.e., affiliative and argumentative conversations) separately.

### 4.1. *Post-hoc* analyses of interaction terms

Results for the standardized models exploring the complex interaction term are presented in Table 2. For clarity, we again refer in the text only to the model variables so that readers can find the relevant statistics in the model. As with the first model, we ran both standardized and unstandardized versions of these models, but we present only the standardized models in the text. Additional information—including the unstandardized models—can be found in the OSF and GitHub repositories for the project (see Section 3).

**Table 2:** Results from two standardized linear mixed-effects models (implemented with lme4; Bates et al., 2015).

**Conv**.	**Predictor**	**Estimate**	**Std. Error**	***t*-value**	***p*-value**	**Sig**.
Aff.	Task	−0.251	0.180	−1.394	0.163	
	LL	0.050	0.072	0.698	0.480	
	QL	0.013	0.056	0.225	0.820	
	LL × QL	−0.044	0.009	−4.782	<0.001	^***^
	Task × LL	0.015	0.072	0.211	0.830	
	Task × QL	−0.054	0.056	−0.973	0.330	
	Task × LL × QL	−0.004	0.009	−0.436	0.660	
Arg.	Task	0.054	0.186	0.290	0.770	
	LL	−0.015	0.060	−0.255	0.800	
	QL	0.132	0.060	2.219	0.026	^*^
	LL × QL	−0.052	0.007	−6.987	<0.001	^***^
	Task × LL	−0.077	0.060	−1.287	0.198	
	Task × QL	0.127	0.051	2.488	0.013	^*^
	Task × LL × QL	−0.005	0.007	−0.622	0.530	

To follow up on the four way interaction term in the main model (see Table 1), we targeted each conversation type in separate models, using their own standardized datasets. The affiliative model's fixed effects alone accounted for 7% of the variance (marginal R^2^ = 0.07), while the fixed and random effects accounted for 91% of the variance (conditional R^2^ = 0.91). The argumentative model's fixed effects alone accounted for 5% of the variance (marginal R^2^ = 0.05), while the fixed and random effects accounted for 94% of the variance (conditional R^2^ = 0.94). ^.^p < 0.10;

* p < 0.05;

^**^p < 0.01;

*** *p < 0.001*.

As in the main model, both follow-up models showed that head movement showed turn-taking patterns with some leader-follower dynamics (*LL* × *QL*). No other effects reached significance in the *post-hoc* analyses of the affiliative conversations.

The results of the *post-hoc* analyses of argumentative conversations, however, showed context-sensitive responses to low-level constraints. Overall, participants demonstrated a much stronger turn-taking pattern of head movement during argumentative conversations (*QL*). These effects were much more pronounced during the dual-task condition than in the noise condition (*task* × *QL*), with recurrence exhibiting the characteristic U-shaped DRP of turn-taking behavior.

### 4.2. Comparisons to phase-randomized baseline

The patterns outlined above hold even compared to baseline measures of synchrony. For brevity, tables of results comparing real data to phase-randomized surrogate baseline data are included in **Appendix**. Table A1 is the companion to Table 1; Table A2 is the companion to Table 2. In these tables, the “data” variable refers to either the baseline surrogate data (*data* = −0.5) or the real experimental data (*data* = 0.5).

Again, only the standardized models are presented in the text of the current paper. Unstandardized models—along with standardized and unstandardized models performed with the sample-wise shuffled baseline—are available on the project's GitHub and OSF repositories (see Section 3). Due to the complexity of the overall model (Table A1), we use the *post-hoc* models (Table A2) as a framework for discussing the results.

#### 4.2.1. Affiliative conversation *post-hoc* analyses for comparison to baseline

Strikingly, these results suggested that the level of recurrence observed in the affiliative conversations was not overall significantly different from baseline (*data*), although the two datasets did differ in their dynamics (*data* × *LL* × *QL*). The surrogate data showed significantly lower leading-following patterns (*data* × *LL*) and exhibited no turn-taking nor synchrony patterns (*data* × *QL* significant, but not *QL*).

The affiliative conversations also differed from baseline with the task data. The results suggested a trend toward significantly higher overall recurrence in the noise condition than we would expect to see by chance, although it did not reach significance (*data* × *task*). We did, however, find a significant difference in the coordination dynamics between the two task conditions (*data* × *task* × *QL*): Compared to the flat recurrence profile of the baseline in both conditions, the dual-task condition demonstrated more of the inverted-U-shape of synchrony, while the noise condition demonstrated more of the U-shape of turn-taking.

#### 4.2.2. Argumentative conversation *post-hoc* analyses for comparison to baseline

Unlike the affiliative conversations, we found that levels of recurrence were—overall—significantly lower than baseline (*data*). In other words, participants coordinated with one another even *less* than what would be expected by chance, and that decreased recurrence was more likely to appear in a turn-taking pattern (*data* × *QL*) with some leader-follower effects (*data* × *LL*).

Task constraints also exerted significant effects on the dynamics of recurrence. In addition to showing different leader-follower behaviors across the two tasks (*data* × *task* × *LL*), the data revealed differences in the temporal patterning of movement across the two tasks (*data* × *task* × *QL*). Essentially, participants' head movements showed much stronger turn-taking patterns in the argumentative conversations in the dual-task condition than in the noise condition, which had a relatively flat recurrence profile.

## 5. Discussion

Communication is a rich, complex phenomenon that plays a central role in daily human life. We use conversation flexibly, allowing us to engage in mundane transactions, bond over shared interests, collaborate to complete joint tasks, and argue about our political opinions. Although these different communicative contexts are part of our everyday experiences, the scientific study of these dynamics have largely centered on friendly or collaborative contexts. The current study aimed to contribute to a fuller picture of communicative dynamics by investigating how conflict (a high-level contextual perturbation) and rapidly changing visual information (a low-level contextual perturbation) interact to affect the dyadic system.

Here, we specifically targeted *interpersonal synchrony* of head movement—that is, the similarity of participants' head movement over time during their interaction. We used PsyGlass (Paxton et al., 2015), a stimulus-presenting and movement-recording application on Google Glass, to capture the acceleration time series of participants' head motion during naturalistic conversations shaped by high-level (i.e., argumentative or affiliative conversational context) and low-level (i.e., noise or dual-task visual information condition) constraints. From the theoretical position that human interaction is a complex adaptive system, we hypothesized that interaction dynamics should be sensitive to each of these constraints.

Our analyses found support for some—but not all—of our hypotheses. Taken together, our results support the idea of interaction as a complex adaptive system while highlighting inconsistencies within previous literature and suggesting avenues for future research.

### 5.1. Head movement synchrony

Perhaps most unexpectedly, we did not find support for our hypothesis that participants would be synchronized in their head movement patterns (*H*_1_). Instead, participants' head movement tended to exhibit time-lagged synchrony or turn-taking dynamics (cf. Butler, 2011). These results stand in contrast with previous work on head movement synchrony, which has shown that individuals tend to synchronize their head movements during conversation.

Interestingly, these patterns resemble those observed in speech signals during friendly and argumentative conversations (Paxton and Dale, 2013c). Of course, this suggests that the current measure of head movement may be influenced by speaking. Future work should disentangle the ways that intrapersonal coupling of head movement and speaking may influence interpersonal head movement coordination.

However, relatively little research has targeted head movement synchrony, and the existing work in this area has used very different methods and analyses. For example, Boker et al. (2002) (a) tracked head movements with passive three-dimensional motion-tracking sensors at 80 Hz, (b) analyzed Euclidean velocity, (c) did not mention whether a filter was used on the movement time series, (d) calculated synchrony through windowed cross-correlation (i.e., a linear time series analysis) and (e) did not use a baseline. On the other hand, Ramseyer and Tschacher (2014) (a) tracked head movements through video (i.e., by quantifying displaced pixels from frame to frame in a region of interest around the head) at an unspecified sampling rate, (b) analyzed a “flattened” velocity (i.e., 2D projection of 3D movement), (c) filtered movement signals with an unspecified filter, (d) calculated synchrony as the absolute value of the windowed cross-correlation coefficients between participants, and (e) used a “window-wise” shuffled baseline (i.e., preserving local structure within the data by shuffling 1-min chunks rather than shuffling all samples independently). By contrast, we (a) tracked head movements with active head-mounted sensors at 10 Hz (after downsampling), (b) analyzed Euclidean *acceleration*, (c) filtered movement signals with a low-pass Butterworth filter, (d) calculated synchrony with cross-recurrence quantification analysis (i.e., a nonlinear time series analysis without windowing), and (e) used a phase-randomized baseline (and, in our Supplementary Materials, a sample-wise shuffled baseline). Future work should explore the degree to which these and other factors may influence findings of head movement synchrony. Our task also differed by integrating high- and low-level constraints. We turn to these next.

### 5.2. Differences in high-level contextual constraints

Conversational context modulated these patterns of coordination (supporting *H*_2_). Consistent with previous research (Paxton and Dale, 2013a), we also found support for our directional hypothesis. Argument decreased synchrony (supporting *H*_2*A*_): Participants moved in more dissimilar ways during argumentative conversations relative to affiliative ones.

The way in which the two high-level contexts influenced synchrony was particularly interesting. Synchrony during friendly conversations was indistinguishable from chance, while synchrony during argumentative arguments was significantly *lower* than what would be expected by chance. This contrasted with our previous work (Paxton and Dale, 2013a), which found that overall body movement synchrony during friendly conversations was higher than expected by chance and that synchrony during arguments was not significantly different than chance. However, this again would be consistent with the patterns observed in speech rather than movement (Paxton and Dale, 2013c), as mentioned earlier.

### 5.3. Differences in low-level contextual constraints

We also found that low-level contextual constraints influenced coordination dynamics (supporting *H*_3_), but the results surrounding our directional hypothesis were more nuanced (*H*_3*A*_). We found no significant differences in the overall levels of synchrony in the presence of informative or uninformative visual input, instead finding differences in the moment-to-moment dynamics of coordination across high-level contextual constraints. The effects of task condition emerged only during arguments, again supporting the idea that emergent behaviors—like synchrony—are context-dependent: Head movements exhibited a marked turn-taking pattern during argumentative conversations in the dual-task condition but had relatively flat temporal correspondence in the noise condition.

In finding an interaction effect for the low-level contextual constraint, the current study may highlight the importance of the cognitive interpretation of the perceptual information in the environment. Previous work on auditory perceptual information simply introduced a noisy background stimulus (Boker et al., 2002); no additional interpretation was needed. The current work, by contrast, presented the same perceptual stimulus to participants (i.e., changing blue and red screens), and the two conditions differed by the significance (or lack thereof) of that stimulus.

Although we found no main effect between task conditions (see Section 5.5), the differences of these two conditions relative to one another can meaningfully inform some of our understanding of these phenomena. The turn-taking coordination dynamics during arguments in the dual-task condition (compared with the flat profile in the noise condition; see Figure 1) may suggest a slight reworking of the influences seen in previous work. The auditory noise of Boker et al. (2002) would have presented task-relevant difficulties, since hearing and speaking are directly affected by ambient noise. By contrast, our “noise” condition—a flashing screen—may not have directly impacted conversation, compared with the increased cognitive load of performing a working memory task while having a complex conversation. This suggests a slight change in what may boost coordination: Like the auditory noise of Boker et al. (2002) and the dual-task condition of the present study, perhaps constraints must be *task-relevant* in order to influence movement coordination.

### 5.4. Conversation as a complex system

The partial support for *H*_3*A*_ provided the strongest evidence for context sensitivity of conversation to high- and low-level constraints. Our results both supported *and* failed to support our directional hypothesis, *depending on the context*. The effects of high- and low-level contextual constraints were neither uniform nor additive; instead, high- and low-level contextual effects interacted to produce unique patterns. We interpret these results as fitting with the idea that conversation can be fruitfully conceptualized through dynamical systems theory (DST), supporting our final hypothesis (*H*_4_).

While previous work explored only perceptual noise within “free conversation” (p. 350; Boker et al., 2002), the present study asked participants to engage in two distinct discourse activities—argument or friendly conversation. This allowed us not only to explore the effects of low-level contextual constraints in a new modality (i.e., vision) but provided us with an opportunity to combine it with a growing emphasis on exploring coordination dynamics in different conversational contexts.

Our results add nuance to previous findings about perceptual noise: Rather than uniformly increasing coordination (cf. Boker et al., 2002), low-level contextual pressures alter coordination dynamics only in some conversational contexts. Our results also add nuance to previous findings about conversational context: Rather than uniformly decreasing synchrony (Paxton and Dale, 2013a; Abney et al., 2014), argument's effects can be modulated by low-level perturbations. Moreover, these low-level perturbations affect behavior differently depending on the overarching high-level context—exerting a stronger influence on coordination dynamics during argument compared to affiliative conversations. Most strikingly, this particular combination of high- and low-level context has led to unique behavioral dynamics, leading both synchrony in both friendly and argumentative conversations to decrease (relative to chance) and to reorganize their temporal dynamics.

Of the contributions of the current study, we believe that our results most compellingly speak to the importance of recognizing conversation as a complex dynamical system. Consistent with the *interpersonal synergies* perspective on coordination (e.g., Riley et al., 2011), we find that coordination is sensitive to contextual constraints. Put simply, coordination—as one property of interaction, which we view as a complex dynamical system—is simultaneously sensitive to low-level perceptual information, cognitive interpretation of this low-level information, and high-level interpersonal goals.

### 5.5. Limitations and future directions

The current paper provides one of the first simultaneous explorations of high- and low-level contextual constraints in naturalistic conversation. As a result, the study has several limitations that are opportune areas for future directions.

First, we found that the difference in recurrence between affiliative and argumentative conversations was modulated by task: Argumentative conversations were more strongly affected by task condition than affiliative conversations (see Table 2). However, this pattern could have emerged in a variety of ways: For example, compared to non-visually-disrupted conversation, noise could have decreased coordination; the dual-task condition could have increased coordination; both could have decreased, with noise simply leading to a greater decrease; both could have increased, with dual-task simply leading to a greater increase; or some other pattern may be at work. Simply put, although we can address *relative* differences between the two conditions, we cannot make strong claims as to the precise mechanism behind the differences in *absolute* coordination from the current study. Future work should include a baseline condition without any visual noise (holding all other experimental pressures equal) in order to target these possibilities. (A baseline condition would also help choose among similar causes behind the difference in peakedness between noise and dual-task conditions in argument.)

**Figure 2:**
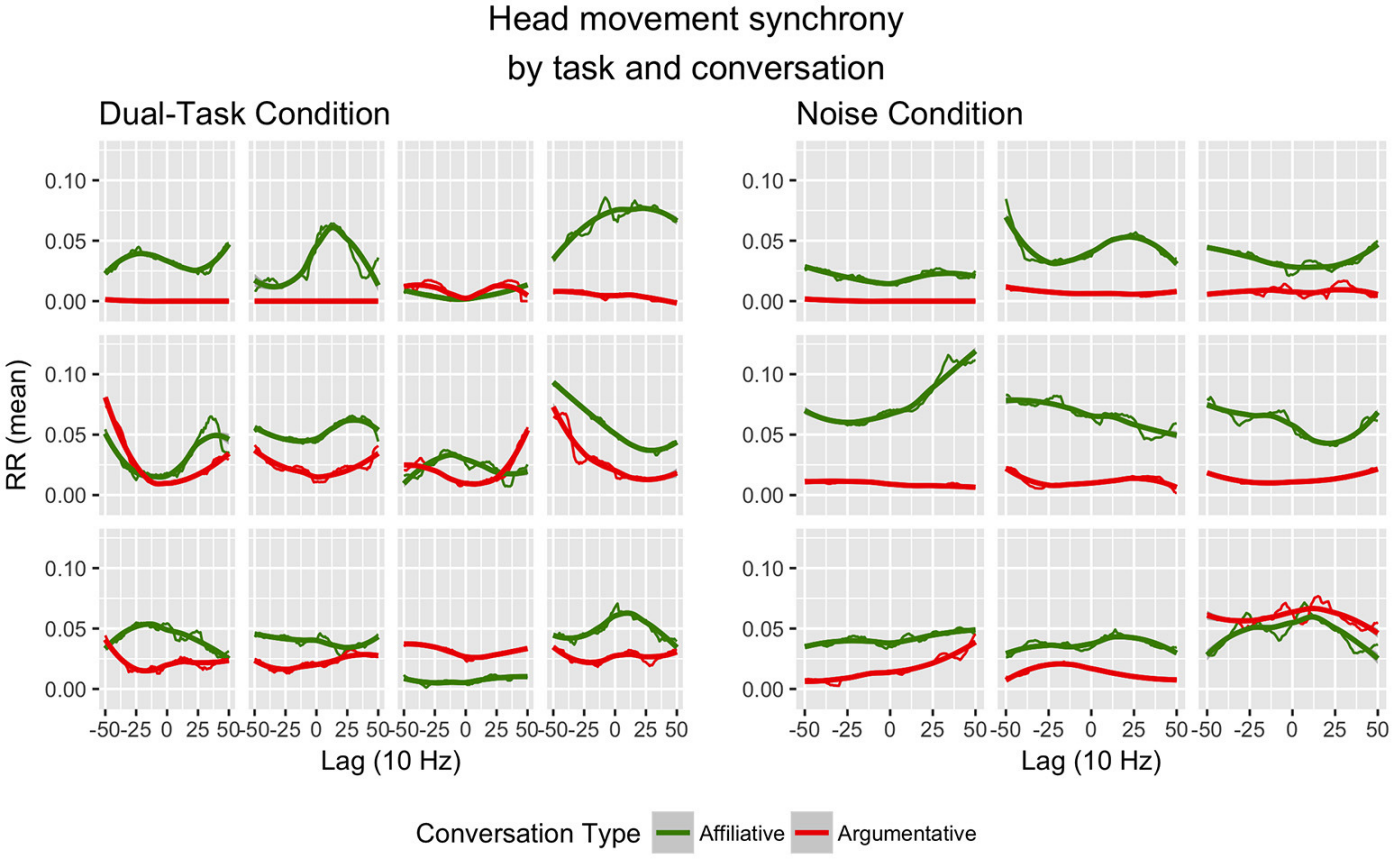
Individual profiles of head movement synchrony for each dyad, divided by conversation type (green = affiliative; red = argumentative) and task condition (left = dual-task; right = noise). Lag is graphed in the 10 Hz sampling rate (i.e., 10 samples per second). Created in R (R Core Team, 2016) with ggplot2 (Wickham, 2009).

Second, we here only investigated linear (i.e., leading/following) and quadratic (i.e., synchrony or turn-taking) patterns across all dyads. As we have observed in our previous work, these data appear to exhibit interesting dyad-specific effects (see Figure 2), and future work should investigate them as dyad-level analogs to individual differences. It may be of interest to include higher-order polynomial patterns (e.g., cubic, quartic) in future analyses, both in describing the observed data and in understanding what they might mean psychologically or interpersonally.

Third, research should continue across additional modalities and contexts. Not all constraints should affect conversation equally; therefore, there should be no expectation that the same dynamics will emerge across all modalities. The effect of low-level constraints in a joint task-performance environment may be quite different than naturalistic conversation. Similarly, introducing perturbations of varying severity to different perceptual modalities may unequally affect interpersonal dynamics. Future work should continue to map out these effects to better understand interaction.

Finally, we present only a first exploration of these dynamics; our findings should be replicated, especially in larger samples. The sample included here is fairly normative for conversational coordination research (for discussion of sample sizes, see Paxton and Dale, 2013a); the only other study exploring the effects of perceptual perturbations on conversation dynamics (to the authors' knowledge) included only 4 dyads (Boker et al., 2002). Issues of open science and reproducibility are particularly salient at this time to psychology and cognitive science (cf. Open Science Collaboration, 2015), so we provide (1) open-source code for our data collection techniques (on the PsyGlass GitHub repository: http://www.github.com/a-paxton/PsyGlass), (2) a high level of methodological detail about our procedure (in Section 2.2), (3) our data (on OSF: https://osf.io/4yqz8/), and (4) open-source code for our data preparation and analysis techniques (on OSF, https://osf.io/4yqz8/, and GitHub, https://www.github.com/a-paxton/dual-conversation-constraints). These tools will help us and other researchers interested in interpersonal coordination and communication dynamics to integrate our practices, resources, and findings so that we can—together—better refine our understanding of human social behavior.

## 6. Conclusion

In this paper, we explore the dynamics of human interaction in an experiment and analyses inspired by ideas from complex adaptive systems. Patterns of nonverbal behavior during conversation change based on both high-level contextual constraints—like what kind of conversation people are having—and low-level contextual constraints—like the significance of visual information in the environment. Replicating previous work, we find that argument decreases movement synchrony. Interestingly, we find that high-level constraints interact with low-level ones, mitigating or exacerbating the effects of argument depending on the cognitive interpretation of the perceptual stimuli. We see our results as contributing to the growing view that patterns of communication—even subtle signatures of body movement—are shaped by the host of contextual factors that surround the conversation.

## Author contributions

Experiment design: AP and RD. Data collection: AP. Data analysis: AP and RD. Manuscript writing and editing: AP and RD.

### Conflict of interest statement

The authors declare that the research was conducted in the absence of any commercial or financial relationships that could be construed as a potential conflict of interest.

## Footnotes

^1^It is outside the scope of the current article to outline the differences in these terms. For more on terminology within this domain, see Paxton and Dale (2013b) and Paxton et al. (2016).

^2^We recognize that this “top” vs. “bottom” categorization is a simplification, as it likely approximates a spectrum of spatial or temporal scales; we nevertheless feel this organizing scheme is useful for emphasizing the differing role of either end of this spectrum.

^3^By “informative” we mean to say simply that participants must attend to the stimulus for a secondary task. We do not mean that the stimulus will be informative for the conversation.

^4^A subset of the affiliative conversations across both between-dyads conditions served as a brief proof-of-concept study in an earlier methodological paper (Paxton et al., 2015). Only the affiliative conversation of dyads who were assigned to the affiliative-first conversation order were included in that analysis.

^5^Described to the participants as “gay and lesbian marriage.”

^6^Participants were also video- and audio-recorded with separate equipment, but this information is not under consideration in the current paper.

^7^Although Main et al. (2016) present the categorical case, the same principles apply to the continuous case, which we employ here.

^8^We do recognize that there are open questions about how to best handle phase space reconstruction for RQA that can extend to CRQA, particularly with regard to the choice of embedding dimension (e.g., Marwan et al., 2007). While some previous work suggests that an embedding dimension of 1 (*m* = 1) is sufficient, we follow current recommendations to determine embedding dimension with false nearest neighbors for each participant in the dyad (Riley and Van Orden, 2005) and selecting the higher embedding dimension of the two (Marwan et al., 2007).

^9^We thank a reviewer for suggesting this more robust analysis.

## Appendix

### Comparisons to baseline

This appendix provides the results of analyses comparing the real experimental data to the phase-randomized surrogate baseline data. Table A1 compares the real and baseline data using the analysis scheme provided in Section 2.4. Table A2 provides *post-hoc* analyses diving into the differences between the affiliative and argumentative conversations.

**Table A1:** Results from the standardized linear mixed-effects model comparing the real data to the phase-randomized surrogate baseline (implemented with lme4; Bates et al., 2015).

**Predictor**	**Estimate**	**Std. Error**	***t*-value**	***p*-value**	**Sig**.
Data Type	−0.133	0.003	−40.421	<0.001	***
Conversation	−0.220	0.128	−1.718	0.0860	.
Task	0.034	0.086	0.396	0.6900	
LL	0.024	0.008	2.894	0.0040	**
QL	0.025	0.006	4.395	<0.001	***
Data × LL	−0.006	0.008	−0.769	0.4400	
Data × QL	0.025	0.009	2.800	0.0050	**
Conversation × Task	0.090	0.096	0.940	0.3500	
Data × Conversation	−0.320	0.047	−6.875	<0.001	***
Data × Task	−0.114	0.006	−20.019	<0.001	***
Data × Conversation × Task	0.044	0.047	0.950	0.3400	
Task × LL	−0.006	0.008	−0.782	0.4300	
Data × Task × LL	−0.012	0.008	−1.417	0.1570	
Conversation × LL	0.001	0.008	0.142	0.8900	
Data × Conversation × LL	−0.029	0.008	−3.495	<0.001	***
Task × Conversation × LL	−0.019	0.008	−2.270	0.0230	*
Data × Task × Conversation × LL	−0.013	0.008	−1.580	0.1140	
Task × QL	0.006	0.006	1.010	0.3100	
Data × Task × QL	0.012	0.009	1.338	0.1810	
Conversation × QL	0.022	0.006	3.772	0.0002	***
Data × Conversation × QL	0.017	0.006	3.061	0.0020	**
Task × Conversation × QL	0.040	0.006	7.054	<0.001	***
Data × Task × Conversation × QL	0.025	0.006	4.454	<0.001	***
LL × QL	−0.026	0.008	−3.129	0.0020	**
Data × LL × QL	−0.011	0.008	−1.328	0.1840	
Task × LL × QL	−0.005	0.008	−0.600	0.5500	
Data × Task × LL × QL	0.002	0.008	0.197	0.8400	
Conversation × LL × QL	0.013	0.008	−1.541	0.1230	
Data × Task × LL × QL	0.015	0.008	1.882	0.0600	.
Conversation × Task × LL × QL	0.004	0.008	0.465	0.6400	
Data × Conversation × Task × LL × QL	−0.004	0.008	−0.429	0.6700	

The model's fixed effects alone accounted for 8% of the variance (marginal R^2^ = 0.08), while the fixed and random effects accounted for 51% of the variance (conditional R^2^ = 0.51). ^.^p < 0.10;

* p < 0.05;

** p < 0.01;

*** *p < 0.001*.

**Table A2:** Results from two standardized linear mixed-effects models comparing real data to phase-randomized surrogate baseline (implemented with lme4; Bates et al., 2015).

**Conv**.	**Predictor**	**Estimate**	**Std. Error**	***t*-value**	***p*-value**	**Sig**.
Aff.	Data	0.062	0.061	1.021	0.310	
	Task	−0.079	0.131	−0.605	0.550	
	LL	0.027	0.030	0.899	0.370	
	QL	0.004	0.009	0.491	0.620	
	Data × LL	0.027	0.013	2.145	0.032	^*^
	Data × QL	0.009	0.016	0.583	0.560	
	Data × Task	−0.191	0.105	−1.820	0.069	.
	Task × LL	0.015	0.030	0.486	0.630	
	Task × QL	−0.042	0.009	−4.755	<0.001	^***^
	Data × Task × LL	0.002	0.013	0.128	0.900	
	Data × Task × QL	−0.016	0.016	−1.044	0.300	
	LL × QL	−0.016	0.013	−1.249	0.212	
	Data × LL × QL	−0.032	0.013	−2.526	0.012	^*^
	Task × LL × QL	−0.011	0.013	−0.838	0.400	
	Data × Task × LL × QL	0.006	0.013	0.493	0.620	
Arg.	Data	−0.276	0.044	−6.282	<0.001	^***^
	Task	0.099	0.112	0.882	0.380	
	LL	0.022	0.019	1.139	0.260	
	QL	0.041	0.012	3.301	0.001	^**^
	Data × LL	−0.030	0.010	−3.015	0.003	^**^
	Data × QL	0.037	0.007	5.262	<0.001	^***^
	Data × Task	−0.061	0.076	−0.800	0.420	
	Task × LL	−0.022	0.019	−1.145	0.250	
	Task × QL	0.040	0.012	3.259	0.001	^**^
	Data × Task × LL	−0.021	0.010	−2.119	0.034	^*^
	Data × Task × QL	0.033	0.007	4.630	<0.001	^***^
	LL × QL	−0.033	0.010	−3.303	0.001	^**^
	Data × LL × QL	0.004	0.010	0.392	0.700	
	Task × LL × QL	−0.001	0.010	−0.095	0.920	
	Data × Task × LL × QL	−0.002	0.010	−0.164	0.870	

To follow up on the interaction terms in the main model (see Table A1), we targeted each conversation type in separate models, using their own standardized datasets. The affiliative model's fixed effects alone accounted for 2% of the variance (marginal R^2^ = 0.02), while the fixed and random effects accounted for 42% of the variance (conditional R^2^ = 0.42). The argumentative model's fixed effects alone accounted for 10% of the variance (marginal R^2^ = 0.10), while the fixed and random effects accounted for 64% of the variance (conditional R^2^ = 0.64).^.^p < 0.10;

* p < 0.05;

** p < 0.01;

*** *p < 0.001*.

## References

[B1] Abney D. H. Paxton A. Dale R. Kello C. T. (2014). Complexity matching in dyadic conversation. J. Exp. Psychol. Gen. 143:2304 10.1037/xge0000021 25285431

[B2] Abney D. H. Paxton A. Dale R. Kello C. T. (2015). Movement dynamics reflect a functional role for weak coupling and role structure in dyadic problem solving. Cogn. Process. 16, 325–332. 10.1007/s10339-015-0648-2 25757891

[B3] Anderson N. C. Bischof W. F. Laidlaw K. E. Risko E. F. Kingstone A. (2013). Recurrence quantification analysis of eye movements. Behav. Res. Methods 45, 842–856. 10.3758/s13428-012-0299-5 23344735

[B4] Barr D. J. (2014). Perspective Taking and Its Impostors in Language Use: Four Patterns. New York, NY: Oxford University Press.

[B5] Barr D. J. Levy R. Scheepers C. Tily H. J. (2013). Random effects structure for confirmatory hypothesis testing: keep it maximal. J. Mem. Lang. 68, 255–278. 10.1016/j.jml.2012.11.001 24403724 PMC3881361

[B6] Barton S. (1994). Chaos, self-organization, and psychology. Amer. Psychol. 49, 5–14. 10.1037/0003-066X.49.1.5 8122818

[B7] Bates D. Mächler M. Bolker B. Walker S. (2015). Fitting linear mixed-effects models using lme4. J. Stat. Softw. 67, 1–48. 10.18637/jss.v067.i01

[B8] Blohowiak B. B. Cohoon J. de Wit L. Eich E. Farach F. J. Hasselman F. (2016). Badges to Acknowledge Open Practices. Available online at: http://osf.io/tvyxz (Accessed 15 December, 2016).

[B9] Boker S. M. Rotondo J. L. Xu M. King K. (2002). Windowed cross-correlation and peak picking for the analysis of variability in the association between behavioral time series. Psychol. Methods 7, 338–355. 10.1037/1082-989X.7.3.338 12243305

[B10] Brennan S. E. Galati A. Kuhlen A. K. (2010). Two Minds, One Dialog: Coordinating Speaking and Understanding, Vol. 53. Burlington, NJ: Elsevier.

[B11] Butler E. A. (2011). Temporal interpersonal emotion systems: the “TIES” that form relationships. Pers. Soc. Psychol. Rev. 15, 367–393. 10.1177/1088868311411164 21693670

[B12] Coco M. I. Dale R. (2014). Cross-recurrence quantification analysis of categorical and continuous time series: an R package. Front. Psychol. 5:510. 10.3389/fpsyg.2014.00510 25018736 PMC4073592

[B13] Coleman P. T. Vallacher R. R. Nowak A. Bui-Wrzosinska L. (2007). Intractable conflict as an attractor: a dynamical systems approach to conflict escalation and intractability. Amer. Behav. Sci. 50, 1454–1475. 10.1177/0002764207302463

[B14] Condon W. S. Sander L. W. (1974). Neonate movement is synchronized with adult speech: interactional participation and language acquisition. Science 183, 99–101. 10.1126/science.183.4120.99 4808791

[B15] Dale R. Fusaroli R. Duran N. Richardson D. C. (2013). The self-organization of human interaction. Psychol. Learn. Motiv. 59, 43–95. 10.1016/B978-0-12-407187-2.00002-2

[B16] Dale R. Kirkham N. Richardson D. (2011). The dynamics of reference and shared visual attention. Front. Psychol. 2:355. 10.3389/fpsyg.2011.00355 22164151 PMC3230789

[B17] Dale R. Spivey M. J. (2006). Unraveling the dyad: using recurrence analysis to explore patterns of syntactic coordination between children and caregivers in conversation. Lang. Learn. 56, 391–430. 10.1111/j.1467-9922.2006.00372.x

[B18] Eckmann J.-P. Kamphorst S. O. Ruelle D. (1987). Recurrence plots of dynamical systems. Europhys. Lett. 4:973.

[B19] Engbert R. Nuthmann A. Richter E. M. Kliegl R. (2005). Swift: a dynamical model of saccade generation during reading. Psychol. Rev. 112, 777–813. 10.1037/0033-295X.112.4.777 16262468

[B20] Fusaroli R. Konvalinka I. Wallot S. (2014). Analyzing social interactions: the promises and challenges of using cross recurrence quantification analysis, in Translational Recurrences, eds Marwan N. Riley M. Giuliani A. Webber C. Jr. (Cham: Springer), 137–155.

[B21] Fusaroli R. Tylén K. (2016). Investigating conversational dynamics: interactive alignment, interpersonal synergy, and collective task performance. Cogn. Sci. 40, 145–171. 10.1111/cogs.12251 25988263

[B22] Garcia C. A. (2015). nonlinearTseries: Nonlinear Time Series Analysis. R Package Version 0.2.3.

[B23] Gewin V. (2016). Data sharing: an open mind on open data. Nature 529, 117–119. 10.1038/nj7584-117a 26744755

[B24] Gibson E. Adolph K. Eppler M. A. (1999). Affordances. Cambridge, MA: MIT Press.

[B25] Gorman J. C. Cooke N. J. Amazeen P. G. Fouse S. (2012). Measuring patterns in team interaction sequences using a discrete recurrence approach. Hum. Factors 54, 503–517. 10.1177/0018720811426140 22908675

[B26] Haken H. (1977). Synergetics. Phys. Bull. 28, 412–414. 10.1088/0031-9112/28/9/027

[B27] Haken H. (1990). Synergetics as a Tool for the Conceptualization and Mathematization of Cognition and Behaviour — How Far Can We Go? Berlin; Heidelberg: Springer.

[B28] Haken H. Portugali J. (1996). Synergetics, Inter-Representation Networks and Cognitive Maps. Dordrecht: Springer.

[B29] Hove M. J. Risen J. L. (2009). It's all in the timing: interpersonal synchrony increases affiliation. Soc. Cogn. 27:949 10.1521/soco.2009.27.6.949

[B30] Iwanski J. S. Bradley E. (1998). Recurrence plots of experimental data: to embed or not to embed? Chaos 8, 861–871. 12779793 10.1063/1.166372

[B31] Kantz H. Schreiber T. (2004). Nonlinear Time Series Analysis. Cambridge: Cambridge University Press.

[B32] Keith T. (2005). Multiple Regression and Beyond. Boston, MA: Pearson.

[B33] Kenrick D. T. Li N. P. Butner J. (2003). Dynamical evolutionary psychology: individual decision rules and emergent social norms. Psychol. Rev. 110, 3–28. 10.1037/0033-295X.110.1.3 12529056

[B34] Kidwell M. C. Lazarević L. B. Baranski E. Hardwicke T. E. Piechowski S. Falkenberg L.-S.. (2016). Badges to acknowledge open practices: a simple, low-cost, effective method for increasing transparency. PLoS Biol. 14:e1002456. 10.1371/journal.pbio.1002456 27171007 PMC4865119

[B35] Konvalinka I. Xygalatas D. Bulbulia J. Schjødt U. Jegindø E.-M. Wallot S.. (2011). Synchronized arousal between performers and related spectators in a fire-walking ritual. Proc. Natl. Acad. Sci. U.S.A. 108, 8514–8519. 10.1073/pnas.1016955108 21536887 PMC3100954

[B36] Lorenz E. N. (1963). Deterministic nonperiodic flow. J. Atmospher. Sci. 20, 130–141.

[B37] Louwerse M. M. Dale R. Bard E. G. Jeuniaux P. (2012). Behavior matching in multimodal communication is synchronized. Cogn. Sci. 36, 1404–1426. 10.1111/j.1551-6709.2012.01269.x 22984793

[B38] Main A. Paxton A. Dale R. (2016). An exploratory analysis of emotion dynamics between mothers and adolescents during conflict discussions. Emotion 16, 913–928. 10.1037/emo0000180 27148849

[B39] March T. Chapman S. Dendy R. (2005). Recurrence plot statistics and the effect of embedding. Physica D 200, 171–184. 10.1016/j.physd.2004.11.002

[B40] Marwan N. Romano M. C. Thiel M. Kurths J. (2007). Recurrence plots for the analysis of complex systems. Phys. Reports 438, 237–329. 10.1016/j.physrep.2006.11.001

[B41] Mathews K. M. White M. C. Long R. G. (1999). Why study the complexity sciences in the social sciences? Hum. Relat. 52, 439–462.

[B42] Miles L. K. Lumsden J. Richardson M. J. Macrae C. N. (2011). Do birds of a feather move together? group membership and behavioral synchrony. Exp. Brain Res. 211, 495–503. 10.1007/s00221-011-2641-z 21448575

[B43] Mirman D. Dixon J. A. Magnuson J. S. (2008). Statistical and computational models of the visual world paradigm: Growth curves and individual differences. J. Mem. Lang. 59, 475–494. 10.1016/j.jml.2007.11.006 19060958 PMC2593828

[B44] Nagaoka C. Komori M. (2008). Body movement synchrony in psychotherapeutic counseling: a study using the video-based quantification method. IEICE Trans. Inform. Syst. 91, 1634–1640. 10.1093/ietisy/e91-d.6.1634

[B45] Nosek B. A. Alter G. Banks G. Borsboom D. Bowman S. Breckler S.. (2015). Promoting an open research culture. Science 348, 1422–1425. 10.1126/science.aab2374 26113702 PMC4550299

[B46] Open Science Collaboration (2015). Estimating the reproducibility of psychological science. Science 349:aac4716 10.1126/science.aac4716 26315443

[B47] Paxton A. Dale R. (2013a). Argument disrupts interpersonal synchrony. Q. J. Exp. Psychol. 66, 2092–2102. 10.1080/17470218.2013.853089 24303888

[B48] Paxton A. Dale R. (2013b). Frame-differencing methods for measuring bodily synchrony in conversation. Behav. Res. Methods 45, 329–343. 10.3758/s13428-012-0249-2 23055158

[B49] Paxton A. Dale R. (2013c). Multimodal Networks for Interpersonal Interaction and Conversational Contexts. Austin, TX: Cognitive Science Society.

[B50] Paxton A. Dale R. (2017). Dual Conversation Constraints: Data and Code for “Interpersonal Movement Synchrony Responds to High- and Low-Level Conversational Constraints.” 10.17605/OSF.IO/4YQZ8 PMC5532444 28804466

[B51] Paxton A. Dale R. Richardson D. C. (2016). Social Coordination of Verbal and Nonverbal Behaviors. Abington; Oxon; New York: Routledge.

[B52] Paxton A. Rodriguez K. Dale R. (2015). Psyglass: capitalizing on Google Glass for naturalistic data collection. Behav. Res. Methods 47, 608–619. 10.3758/s13428-015-0586-z 25893865

[B53] Pickering M. J. Garrod S. (2004). Toward a mechanistic psychology of dialogue. Behav. Brain Sci. 27, 169–190. 10.1017/S0140525X04000056 15595235

[B54] R Core Team (2016). R: A Language and Environment for Statistical Computing. Vienna: R Foundation for Statistical Computing.

[B55] Ramseyer F. Tschacher W. (2014). Nonverbal synchrony of head-and body-movement in psychotherapy: different signals have different associations with outcome. Front. Psychol. 5:979. 10.3389/fpsyg.2014.00979 25249994 PMC4155778

[B56] Richardson D. C. Dale R. (2005). Looking to understand: the coupling between speakers' and listeners' eye movements and its relationship to discourse comprehension. Cogn. Sci. 29, 1045–1060. 10.1207/s15516709cog0000_29 21702802

[B57] Richardson M. J. Marsh K. L. Isenhower R. W. Goodman J. R. L. Schmidt R. C. (2007a). Rocking together: dynamics of intentional and unintentional interpersonal coordination. Hum. Movement Sci. 26, 867–891. 10.1016/j.humov.2007.07.002 17765345

[B58] Richardson M. J. Schmidt R. C. Kay B. A. (2007b). Distinguishing the noise and attractor strength of coordinated limb movements using recurrence analysis. Biol. Cybern. 96, 59–78. 10.1007/s00422-006-0104-6 16953458

[B59] Riley M. A. Richardson M. Shockley K. Ramenzoni V. C. (2011). Interpersonal synergies. Front. Psychol. 2:38. 10.3389/fpsyg.2011.00038 21716606 PMC3110940

[B60] Riley M. A. Van Orden G. C. (2005). Tutorials in Contemporary Nonlinear Methods for the Behavioral Sciences. Available online at: https://www.nsf.gov/sbe/bcs/pac/nmbs/nmbs.jsp

[B61] Sander L. W. (1975). Infant and caretaking environment investigation and conceptualization of adaptive behavior in a system of increasing complexity, in Explorations in Child Psychiatry, ed Anthony E. J. (Boston, MA: Springer), 129–166.

[B62] Shockley K. Richardson D. C. Dale R. (2009). Conversation and coordinative structures. Top. Cogn. Sci. 1, 305–319. 10.1111/j.1756-8765.2009.01021.x 25164935

[B63] Shockley K. Santana M.-V. Fowler C. A. (2003). Mutual interpersonal postural constraints are involved in cooperative conversation. J. Exp. Psychol. 29, 326–332. 10.1037/0096-1523.29.2.326 12760618

[B64] Squires N. K. Squires K. C. Hillyard S. A. (1975). Two varieties of long-latency positive waves evoked by unpredictable auditory stimuli in man. Electroencephalogr. Clin. Neurophysiol. 38, 387–401. 10.1016/0013-4694(75)90263-1 46819

[B65] Stadler M. Kruse P. (1990). The Self-Organization Perspective in Cognition Research: Historical Remarks and New Experimental Approaches. Berlin; Heidelberg: Springer.

[B66] Theiler J. Eubank S. Longtin A. Galdrikian B. Farmer J. D. (1992). Testing for nonlinearity in time series: the method of surrogate data. Physica D 58, 77–94. 10.1016/0167-2789(92)90102-S

[B67] Tschacher W. Rees G. M. Ramseyer F. (2014). Nonverbal synchrony and affect in dyadic interactions. Front. Psychol. 5:1323. 10.3389/fpsyg.2014.01323 25505435 PMC4241744

[B68] Vallacher R. R. Read S. J. Nowak A. (2002). The dynamical perspective in personality and social psychology. Pers. Soc. Psychol. Rev. 6, 264–273. 10.1207/S15327957PSPR0604_01

[B69] Vallacher R. R. Van Geert P. Nowak A. (2015). The intrinsic dynamics of psychological process. Curr. Direct. Psychol. Sci. 24, 58–64. 10.1177/0963721414551571

[B70] Van Orden G. C. Goldinger S. D. (1994). Interdependence of form and function in cognitive systems explains perception of printed words. J. Exp. Psychol. 20, 1269–1291. 10.1037/0096-1523.20.6.1269 7844512

[B71] Van Orden G. C. Holden J. G. Turvey M. T. (2003). Self-organization of cognitive performance. J. Exp. Psychol. Gen. 132, 331–350. 10.1037/0096-3445.132.3.331 13678372

[B72] Wickham H. (2009). ggplot2: Elegant Graphics for Data Analysis. New York, NY: Springer-Verlag.

